# Theoretical prediction of strain tuneable quaternary spintronic Heusler compounds

**DOI:** 10.1107/S2052252517015299

**Published:** 2017-10-27

**Authors:** Jan-Willem G. Bos

**Affiliations:** a Institute of Chemical Sciences and Centre for Advanced Energy Storage and Recovery, School of Engineering and Physical Sciences, Heriot-Watt University, Edinburgh EH14 4AS, UK

**Keywords:** Heusler materials, spin-gapless semiconductors, band structures, magnetic properties, materials modelling, spintronic technologies

## Abstract

Heusler materials have attracted a large amount of attention in the development of spintronic technologies. In this issue, Wang *et al.* [*IUCrJ* (2017), **4**, 758–768] show how strain can be used to tune the band structure of these materials.

The development of spintronic technologies, where both charge and spin of the electron are used, relies on the development of new magnetic materials (Felser *et al.*, 2007 ▸). To become a working technology, the availability of spin-polarized metals and semiconductors that can operate at room temperature is essential. In this context, Heusler materials have attracted a large amount of attention as they support magnetic ordering temperatures in excess of a 1000 K (Felser *et al.*, 2015 ▸). In addition, the crystal structure is compatible with conventional semiconductors and is extremely flexible with regards to the elements it can accommodate. Historically, the development of magnetic Heuslers has been driven by theory, with the prediction of half-metallic conduction in NiMnSb a key milestone in spintronics (de Groot *et al.*, 1983 ▸). The article on rare-earth-based quaternary Heusler compounds, published in this issue of **IUCrJ**, follows this tradition and predicts rare strain-tuneable electronic band structures that may be exploited in spintronics (Wang *et al.*, 2017 ▸).

Heuslers have the *X*_2_ *YZ* formula with more than a 1000 known compositions (Graf *et al.*, 2011 ▸). *Z* is always a main-group element, and *X* and *Y* are commonly transition metals but *s*-block and lanthanide metals can be incorporated. The crystal structure consists of four interpenetrating face-centred cubic sublattices that are offset along the body diagonal of the unit cell. In the normal Heusler structure, the atoms follow an *XYXZ* sequence, where the *Y* and *Z* atoms form a rock-salt structure and the *X* atoms occupy tetrahedral sites. Wang *et al.* have investigated the quaternary *M*CoV*Z* Heusler compounds (*M* = Lu, Y; *Z* = Si, Ge). This is a special subclass of quaternary Heuslers with the so-called *Y* structure with full ordering of the four metals. This is illustrated in Fig. 1 ▸ with the *M* and *Z* making up a rock-salt structure, and Co and V ordered over the tetrahedral sites. As discussed in Wang *et al.*, Heuslers with the *Y* structure have been investigated previously but compositions with rare-earth metals have so far remained unexplored.

The most striking finding by Wang *et al.* is that strain can be used to tune the band structure of these materials. At their equilibrium lattice parameter, all compositions are magnetic semiconductors (MSs) with band gaps for both spin directions. Application of strain enables the electronic structure to be tuned towards spin-gapless-semiconducting and half-metallic behaviour. The schematic densities of states for half metals (HMs) and spin-gapless semiconductors (SGSs) are illustrated in Fig. 2 ▸. In an HM, one spin direction is metallic, while the other is gapped, leading to spin-polarized electrical transport. This key property for spintronics was recently confirmed experimentally in the Heusler Co_2_MnSi (Jourdan *et al.*, 2014 ▸). SGSs have semiconducting band structures with zero band gap for at least one of the spin channels (Wang, 2008 ▸; Ouardi *et al.*, 2013 ▸). This is illustrated in Fig. 2 ▸ for the two types of SGS found in the *M*CoV*Z* Heuslers. Type I SGSs have zero band gap for one spin direction, while the other is gapped. This enables the generation of spin-polarized electrons and holes at almost no excitation cost, which is beneficial for use in spin filters, where the flow of one spin direction is blocked. Type II SGSs are gapped for the individual spin directions but have zero band gap between the majority valence and minority conduction bands. This arrangement allows the excitation of electron and hole currents of opposing spin polarization. The calculations show that the *M*CoV*Z* samples follow the Slater–Pauling rule and have a net magnetization of 21 – 18 = 3 Bohr magneton. They are therefore ferromagnetic SGSs and HMs.

Two distinct sequences of transitions are observed depending on the nature of the rare earth. For *M* = Lu, transitions from MS → SGS (type II) → HM are found. The samples with *M* = Y show a more complex re-entrant behaviour as the structure goes from compressed to expanded with the following sequence: HM → SGS (type I) → MS → SGS (type II) → HM. For LuCoVGe, a 3% change in lattice parameter covers the sequence of transitions, suggesting that this can be experimentally realised in strained epitaxial films. This is important because it offers a route to manipulate the properties of these materials that does not involve changing their chemical composition.

The challenge is now for experimentalists to realise these materials and test the theoretical predictions. The related Y-structure composition, CrVTiAl, has recently been synthesized and used as a spin filter (Stephen *et al.*, 2016 ▸), suggesting that this may be achievable, despite the well known difficulties in obtaining Heuslers devoid of disorder, and the detrimental impact this has on the SGS state (Galanakis *et al.*, 2014 ▸). Stability calculations in Wang *et al.* suggest that it should be possible to prepare these materials. These are exciting times for magnetic Heusler compounds with theory once again guiding the way to new functional properties.

## Figures and Tables

**Figure 1:**
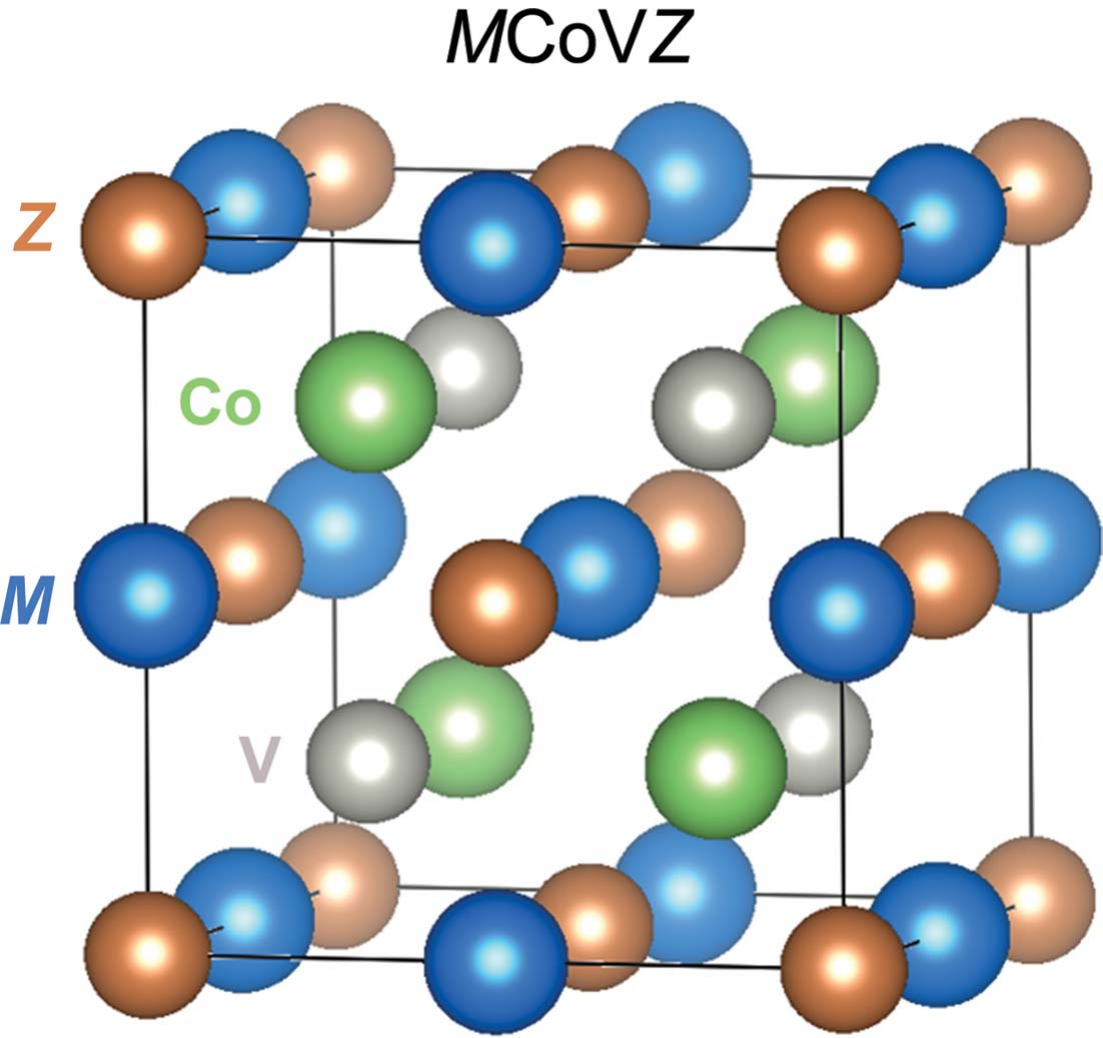
Atomic arrangement for the *M*CoV*Z* Heusler materials studied by Wang *et al.* (2017 ▸) (*M* = Y, Lu; *Z* = Si, Ge).

**Figure 2:**
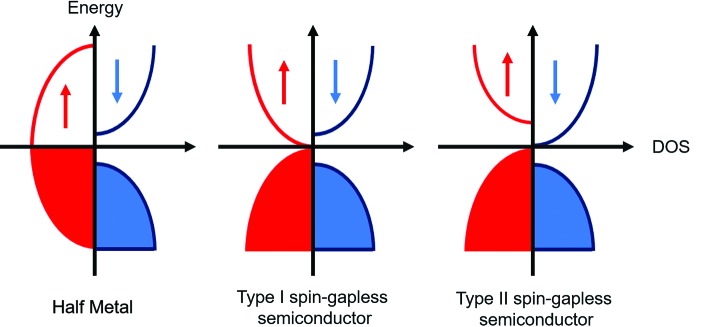
Schematic density of states diagrams for a half metal and the two types of spin-gapless semiconductor found in the *M*CoV*Z* Heuslers studied by Wang *et al*.
